# Use of Automated Ambulatory-Care Encounter Records for Detection of Acute Illness Clusters, Including Potential Bioterrorism Events

**DOI:** 10.3201/eid0808.020239

**Published:** 2002-08

**Authors:** Ross Lazarus, Ken Kleinman, Inna Dashevsky, Courtney Adams, Patricia Kludt, Alfred DeMaria, Richard Platt

**Affiliations:** * Brigham and Women’s Hospital, Harvard Medical School, Boston, Massachusetts, USA; † University of Sydney School of Public Health, Sydney, Australia; ‡ Harvard Pilgrim Health Care and Harvard Vanguard Medical Associates, Boston, Massachusetts, USA; § CDC Eastern Massachusetts Prevention Epicenter and HMO Research Network Center for Education and Research in Therapeutics, Boston, Massachusetts, USA; ¶ Massachusetts Department of Public Health, Boston, Massachusetts, USA

**Keywords:** bioterrorism, surveillance, ambulatory care, statistics

## Abstract

The advent of domestic bioterrorism has emphasized the need for enhanced detection of clusters of acute illness. We describe a monitoring system operational in eastern Massachusetts, based on diagnoses obtained from electronic records of ambulatory-care encounters. Within 24 hours, ambulatory and telephone encounters recording patients with diagnoses of interest are identified and merged into major syndrome groups. Counts of new episodes of illness, rates calculated from health insurance records, and estimates of the probability of observing at least this number of new episodes are reported for syndrome surveillance. Census tracts with unusually large counts are identified by comparing observed with expected syndrome frequencies. During 1996–1999, weekly counts of new cases of lower respiratory syndrome were highly correlated with weekly hospital admissions. This system complements emergency room- and hospital-based surveillance by adding the capacity to rapidly identify clusters of illness, including potential bioterrorism events.

Rapid identification of unusual clusters of acute illness in the general population is a fundamental challenge for public health surveillance (1). Recent distribution of *Bacillus anthracis* spores and the resulting occurrence of clinical disease (2) provide new impetus to developing and implementing surveillance systems that can identify both bioterrorism events and naturally occurring illness clusters, such as influenza and waterborne disease. Recognizing individual cases of infection, e.g., inhalational anthrax, requires astute and alert clinicians. However, many potential biological agents of terrorism, including anthrax, have nonspecific prodromal phases, and no explicit diagnosis is ever made for many other syndromes of potential importance. Recognizing these clusters at the earliest possible opportunity will require well-designed surveillance systems to ensure timely detection of unusual clusters of prodromal, nonspecific illness.

Several projects have been developed specifically to provide improved surveillance for detecting bioterrorism in urban populations (3). Some of these existing surveillance systems operate in emergency departments and hospitals (4). While these systems are very useful, implementation may be impeded by the effort required for timely collection and analysis of diagnosis data in a suitable format. Additionally, emergency rooms and hospitals may see increased numbers of cases days after the first, milder symptoms of disease bring new patients to ambulatory-care settings.

Surveillance systems based in ambulatory-care settings, particularly those based on automated medical records, may therefore provide worthwhile additional information. One of the best-known such systems is the Department of Defense Electronic Surveillance System for the Early Notification of Community-based Epidemics (ESSENCE) system (5), which is based on encounter data from health services operated by the Department of Defense. Another such system is operating in Minnesota (6). Nurse hot lines have also been used for surveillance purposes (7).

We describe here an automated system developed in a partnership between the Centers for Disease Control and Prevention, the Massachusetts Department of Public Health, a large group practice, a health plan, and an academic department. The system produces next-day information about illness clusters, based on ambulatory-care visits and telephone calls.

## Methods

The utility of diagnoses from automated ambulatory encounter data for detecting respiratory disease clusters has been described (8). In this report, we extend the use of encounter data to produce daily surveillance summary reports covering a broad range of syndromes for use by public health officials and health-care providers.

The encounter data come from an electronic medical record system used by Harvard Vanguard Medical Associates, a large multispecialty group practice, to record all ambulatory-care encounters, including telephone contacts, regular visits, and urgent-care encounters, but not emergency room visits. The practice serves approximately 250,000 members, representing approximately 10% of the population of eastern Massachusetts.

The automated record system is a commercial product (Epicare; Epic Systems Corporation, Madison, Wisconsin; available from URL: http://www.epicsys.com) used by many large medical groups. It represents a valuable source of surveillance data because it operates in real time (i.e., records are updated as information is entered). Additionally, to the extent that practices engage in some form of prepaid care, the population served can be explicitly enumerated; the surveillance report described below is restricted to approximately 175,000 members of Harvard Pilgrim Health Care, a principal health maintenance organization in the region. These persons constitute a defined population that receives essentially all its ambulatory care in this practice. Demographic information and addresses are available for all these persons. At the time of consultation, clinical diagnoses are assigned for each encounter by the clinician, who chooses from lists of terms on the encounter screen; essentially all episodes are coded by the end of the same day on which care is given. Although an unlimited number of codes can be chosen, approximately 90% of encounters have three or fewer codes assigned (8), stored as ICD-9 codes. Each night, an extract is created of all encounters recorded in the previous 24 hours with any of >1,500 ICD-9 codes in any of the syndrome categories. The patient's temperature is also recorded along with the ICD-9 codes. Demographic data are merged with each record through a link to the patient’s membership record.

As a way of grouping insured persons into neighborhoods, the addresses of the insured plan members, obtained from the HMO's data, have been coded by Geographic Information System (Mapping Analytics, Rochester, NY) to determine the census tracts of their residences (9,10).

### Developing and Defining Syndromes

Patient encounters are categorized into syndrome groups according to the ICD-9 codes assigned at the time of consultation. The surveillance software considers each encounter record in turn and merges related ICD-9 diagnosis codes into syndrome groups by using a modification of a provisional classification scheme developed as part of the ESSENCE project (5). This scheme reduces the complexity of the ICD-9 into eight syndrome categories: coma/shock, neurologic, upper gastrointestinal, lower gastrointestinal, upper respiratory, lower respiratory, dermatologic, and sepsis/fever. We made two major modifications of the syndrome definitions: the number of ambulatory episodes in the coma/shock category was almost zero in 4 years of data we examined, so it was combined with the neurologic syndrome category. A new syndromic category representing diagnoses of Centers for Disease Control and Prevention (CDC) bioterrorism category A agents (11) (anthrax, botulism, plague, smallpox, tularemia, and hemorrhagic fever) is reported separately. We also added an additional influenza-like illness category, defined by the CDC sentinel surveillance definition of fever >37.8^o^C plus cough and/or sore throat in the absence of a known cause (12).

An individual patient may have multiple encounters associated with a single episode of illness (e.g., initial consultation, consultation 1–2 days later for laboratory results, and follow-up consultation a few weeks later) (8). To avoid double counting from this common pattern of ambulatory care, the first encounter for each patient within any single syndrome group is reported, but subsequent encounters with the same syndrome are not reported as new episodes until >6 weeks has elapsed since the most recent encounter in the same syndrome. We have reported that grouping respiratory illness visits into episodes reduces the total number of events by 38% in this clinical setting (8). This practice of grouping clinical encounters into episodes of illness occurs independently for different syndromes. For example, a patient could qualify for two different syndromes on a single visit if codes for cough (lower respiratory syndrome) and diarrhea (lower gastrointestinal syndrome) are assigned at the same visit; or a lower gastrointestinal syndrome episode could begin a few days after the start of a lower respiratory syndrome episode.

### Reporting Results

**Figure 1:**
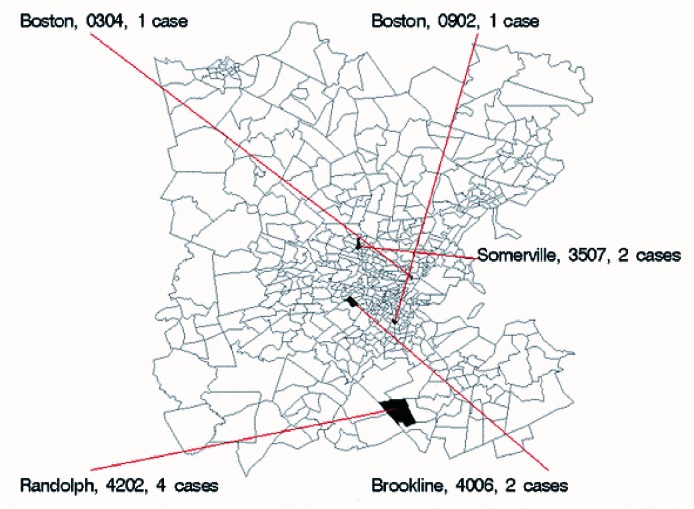
Map of sample small area syndrome counts for Monday, March 4, 2002, showing the five census tracts with the most extreme probability values. Labels show name of town, census tract code (state and county prefixes have been removed), and number of cases for the 24 hours included in the report.

A daily surveillance summary report (Tables 1 and 2; Figure 1) was designed in collaboration with staff from the Massachusetts Department of Public Health and the medical group's administration, which has operated since it was implemented on October 25, 2001. The aim of the report was to identify any unusually large numbers of episodes of illness within the ambulatory-care system. The current version (Table 1) shows new episode counts and rates (per 1,000 insured persons) for all syndromes combined and for each individual syndrome, during the previous day. Mean rates are presented for the same day of the week in the same month of the previous 2 years, as well as the statistical probability associated with these counts derived from a generalized linear mixed model (described in the Models and Analysis section) for the four most common syndromes.

**Table 1:** Daily public health surveillance report of office visits with diagnoses corresponding to infection syndromes: summary report for Monday, March 4, 2002, Massachusetts

Syndrome	Rate/1,000 health plan members (no. of visits)^a^	Probability^b^	1999 average rates for this weekday in the same month	2000 average rates for this weekday in the same month
All combined	2.015 (328)		1.918	2.123
Upper respiratory	1.087 (177)	0.999	1.151	1.251
Lower respiratory	0.405 (66)	0.999	0.369	0.474
Upper gastrointestinal	0.166 (27)	0.999	0.094	0.110
Lower gastrointestinal	0.227 (37)	0.999	0.221	0.173
CNS/neurologic^c^	0.000 (0)		0.003	0.007
Dermatologic	0.012 (2)		0.023	0.022
Sepsis/fever	0.000 (0)		0.057	0.086
Influenza-like illness	0.117 (19)		—	—
CDC bioterrorism category A Agents^d^	0.000 (0)		0	0

^a^Repeated visits within 6 weeks excluded. ^b^Probability of at least this many episodes occurring at least once per year, when the data are adjusted for month, day of week, holidays, secular trend, and variability among census tracts ^c^CNS, central nervous system; CDC, Centers for Disease Control and Prevention. ^d^Anthrax, botulism, plague, smallpox, tularemia, and hemorrhagic fever.

**Table 2:** Lower respiratory syndrome by census tract, Massachusetts: sample small area report for March 4, 2002^a^

Population center	Census tract code	Cases in tract	Denominator in this tract	No. of days between counts this extreme^b^
Randolph	250214202	4	1,232	1
Brookline	250214006	2	730	1
Boston	250250902	1	136	1
Somerville	250173507	2	918	1
Boston	250250304	1	225	1

^a^No census tract had an unusual number of new lower respiratory syndrome episodes on that day. The five most extreme tracts are shown, plus all with counts not expected to occur more than once per month. Tracts with most extreme counts are compared with their own history. ^b^Estimated number of days between counts this extreme in any of the 529 census tracts, when data are adjusted for this tract's unique characteristics, as well as month, day of week, holidays, and secular trend.

Each day's report also includes a list and maps of the residence locations of cases with respiratory and gastrointestinal syndromes (Tables 1 and 2; Figure 1). The list and the map both show the five census tracts in the region with the most improbably large number of new episodes, based on the statistical model described. Daily updates are disseminated to authorized persons through a password-protected area on a Secure Sockets Layer (SSL) (13,14) encrypted website.

#### Models and Analysis

 For each syndrome, we used a generalized linear mixed model (GLMM) (15–17) to model the daily counts from local neighborhoods over a 4-year historical period. In our model, census tracts (CT) form the neighborhoods, but this unit can be extended easily to larger or smaller geographic units if desired. Sample SAS code is provided in Appendix 1. The model closely resembles logistic regression, so that the logit, log(
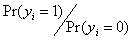
), is modeled as a linear function of some covariates: 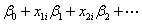
where* i* indexes units of analysis, 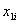
and 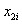 are covariates or predictors, and 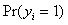 is often denoted as 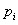 In the GLMM version of logistic regression, 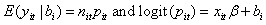 where 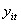
is the binomial-distributed number of visits in CT *i* on day *t*, 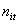 is the number of members living in that CT on that day, 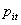 is the probability that any patient has had a visit with a diagnosis in the syndrome, 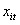 is a set of covariates measured on CT *i* at time *t*, 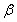 is a vector of fixed effects, and 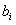 is a random effect distributed with mean 0 and variance 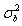. The model can be used to generate an estimate of 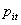 by inverting the logit.

 In the model now in use, we include in 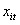 an intercept, an indicator for 6 days of the week, indicators for 11 months of the year, an indicator of the day as regular or a national holiday, and a linear term for the secular time trend. In each case, the terms contribute significantly to the fit of the model (p <0.0001). The estimates from the model have face validity: the estimated odds of visits for lower respiratory infections are higher in winter months than summer, higher on weekdays than on the weekend, and smaller on national holidays. The test that 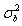
= 0 tests the null hypothesis that all the census tracts are the same, meaning that 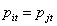
for all CT *i* and *j*. This test is rejected (p <0.0001).

 For example, suppose that the estimated intercept was –8, the estimated effect for April was –0.6, and the estimated effect for Monday was –0.5. Finally, if we are interested in finding 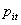 for a given day in April in a CT with an estimated 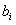 of 1.1, we omit the secular time trend for simplicity. The estimated 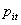, 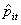, for any Monday in April in that CT is 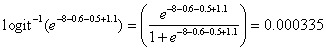.

The models are applied at the CT level to estimate the period of observation required to expect one count at least as high as those observed in each CT for each syndrome, after the data were adjusted for day of week, holidays, season, secular trend, and the unique characteristics of each CT. This period is also corrected to reflect the fact that each CT is considered on each day. The reported period is the inverse of the expected number of counts this extreme in a day, where 529 tests are performed each day. This is 529*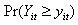 where the last figure is the probability under the model that as many or more cases than were observed on that day will be observed in CT *i*, calculated from the binomial distribution function with 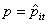 and 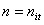. The surveillance report (Table 2) shows the five CTs with the longest required period derived from the model, plus all CTs with counts likely to occur only once a month or less often. We present the model in this fashion so that large numbers are unusual, rather than the smaller-is-more-unusual format of the p-value. In addition, this format has the advantage of being measured in the time scale rather than the probability scale. A map of eastern Massachusetts shows the spatial relationship between CTs highlighted each day (Figure 1).

 We also used the model to generate the probability that a count as large or larger than the observed count would be seen over the whole surveillance area, after adjusting for day of week, holidays, season, and census tract variation. This adjustment is done by the same process as for the individual tracts except that the random effects are omitted. These values are also then adjusted on a yearly basis to account for the fact that the probabilities are estimated every day. This estimate is simply the probability that a count as or more extreme as the observed one would be observed in 365 days. All the statistical processing uses automated SAS (18) programs. The web interface was developed by using the Zope web application platform (19), which runs a Python (20) program to rewrite the SAS output files as linked web pages.

#### Validation

In the absence of known bioterrorism events, one way of validating the surveillance system is to compare the relationship between the substantial seasonal changes in disease incidence known to occur in the ambulatory-care setting (8) to the seasonal pattern in a reliable and independent source of data such as the hospital system. The lower respiratory syndrome includes a range of diseases (8) commonly associated with admission for an acute illness after a variable prodrome, so this syndrome was chosen for comparison.

**Figure 3:**
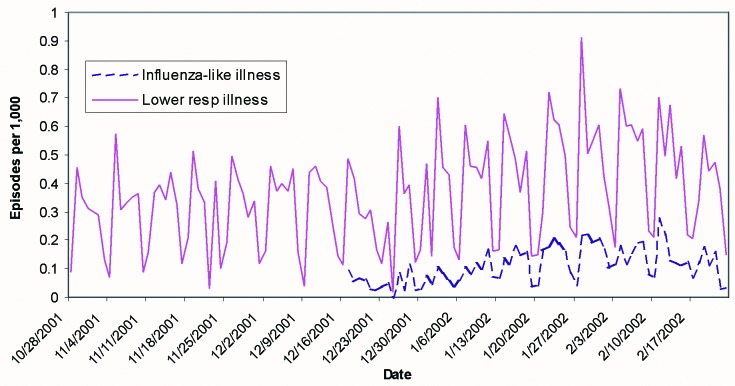
Health plan membership by census tract in eastern Boston. Each census tract contains approximately 4,000 residents.

Health plan membership is not uniformly distributed throughout the population of Massachusetts (Figure 3). One hundred twenty zip codes were identified in which >100 lower respiratory syndrome episodes were identified in health plan members during 1996–1999; these cases accounted for approximately 70% of all ambulatory lower respiratory syndrome episodes recorded in health plan members.

The weekly numbers of these episodes in health plan members were compared with weekly hospital admissions for all residents (not limited to health plan members) of the same 120 zip codes. Hospital admission data with personal identifiers removed were obtained from the Massachusetts Division of Health Care Finance and Policy for the 3 years ending September 30, 1999. These records included only patients discharged from the hospital, so the final 3 weeks of the hospital admission data were truncated to minimize the “edge effect” from the period when patients may have been admitted but not yet discharged and thus were not included in the available data.

Using the same procedure to group hospital discharge ICD-9 codes as was used for the ambulatory data, we identified all admissions from residents of the 120 zip codes who had a discharge diagnosis in the lower respiratory syndrome group. Hospitalizations were assigned to the date of admission. We compiled the number of ambulatory lower respiratory syndrome episodes and the number of hospital admissions for lower respiratory syndrome for each week for the 3 years ending September 30, 1999. Time-series plots were prepared to compare seasonal patterns in the two independent data sources, and Spearman rank correlations were calculated between weekly hospital admission counts and ambulatory care episodes in the same week, the previous week, and so on up to 6 weeks, by using SAS Proc CORR (18).

## Results

**Figure 2:**
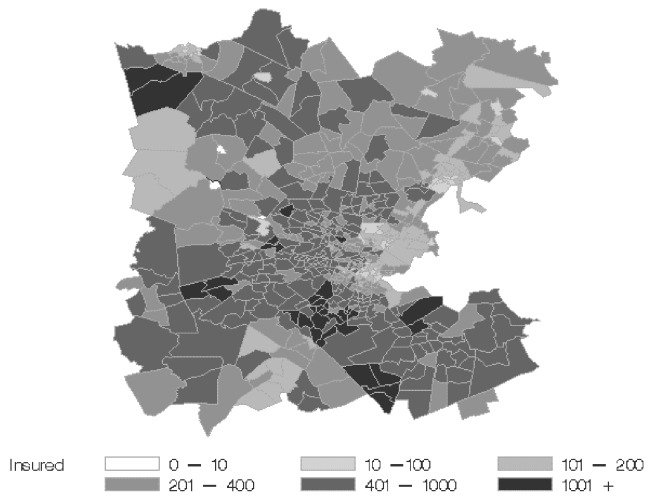
Daily incidence rates of lower respiratory and influenza-like illness after December 17, 2001, showing that within-week variation is substantially greater than seasonal variation.

Data from an example of the summary report, one of the syndrome census tract reports, and the corresponding map for March 4, 2002, are shown in Table 1, Table 2 and Figure 1, respectively. The overall counts were all well within model-based expectations for this time of year, so the associated probabilities were all close to 1; at the level of census tracts, all counts are common enough to be expected daily. Figure 2 shows daily rates of new episodes of influenza-like illness and lower respiratory syndromes. Day-to-day variation is marked, especially on weekends, as is the expected winter increase in rates. Holidays such as New Year’s Day have the lowest rates of reported illness.

The sensitivity of the statistical model in the face of this extreme day-to-day and seasonal variation is illustrated in Table 3. As few as three cases among health plan members may constitute an event predicted by the GLMM to occur less often than once per year, depending on the day of week and the month of the year.

**Table 3:** Number of episodes of lower respiratory syndrome that would be expected to occur only once a month and once a year, based on a generalized linear mixed model (GLMM), in a representative eastern Massachusetts census tract^a^

Month	Day of week	No. needed for once per month event	No. needed for once per year event
January	Monday	5	6
January	Tuesday	5	6
January	Wednesday	5	6
January	Thursday	5	6
January	Friday	5	5
January	Saturday	4	4
January	Sunday	4	4
April	Monday	4	5
April	Tuesday	4	5
April	Wednesday	4	5
April	Thursday	4	5
April	Friday	4	5
April	Saturday	3	4
April	Sunday	3	4
July	Monday	4	5
July	Tuesday	4	4
July	Wednesday	4	4
July	Thursday	4	4
July	Friday	4	4
July	Saturday	3	4
July	Sunday	3	4
October	Monday	5	6
October	Tuesday	4	5
October	Wednesday	4	5
October	Thursday	4	5
October	Friday	4	5
October	Saturday	4	4
October	Sunday	4	4

^a^This census tract has 491 health plan members and a random effect of 0.083, illustrating the effect of day of week and month of year for 2002.

**Figure 4:**
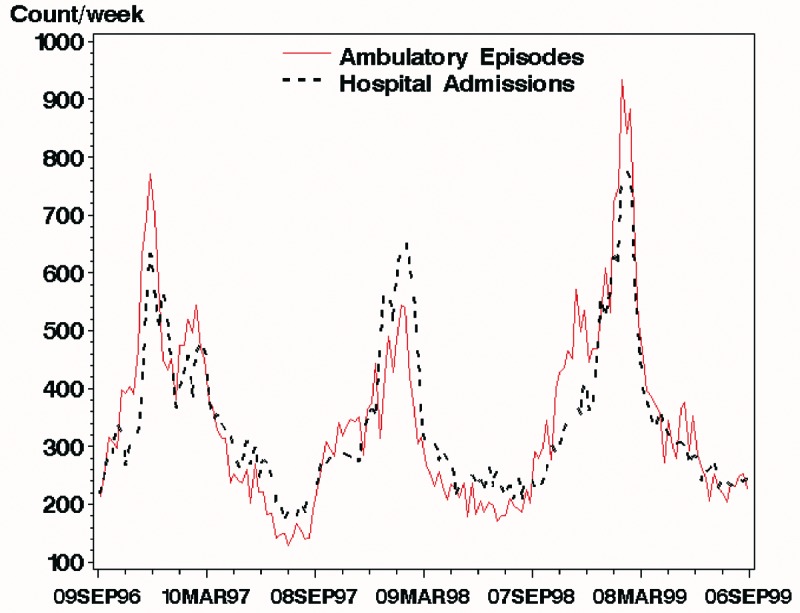
Weekly total ambulatory-care episodes of lower respiratory syndrome (broken line) and hospital admissions for lower respiratory syndrome (solid line) in Massachusetts for the 3 years from September 9, 1996, through September 9, 1999. The eligible population for the hospital data was the entire population of each zip code; the ambulatory care data came from a variable subset of each zip code. As a result, the number of hospital admissions was higher than the number of ambulatory-care episodes for parts of the period shown.

Visual inspection of the weekly counts of episodes in the ambulatory setting compared with hospital admissions shows congruent patterns, including pronounced winter peaks (Figure 4). The data for admissions appear to lag behind data for ambulatory-care visits, most obviously for the winters of 1997 and 1999. Overall, weekly ambulatory-care episodes for lower respiratory illness were highly correlated with hospital admissions over the 3 years examined. The Spearman rank correlation between hospital admissions and ambulatory-care visits during the same week was 0.89. Correlating hospital admissions with ambulatory encounters from the previous week yielded a value of 0.90. Repeating this analysis, increasing the lag by 1 week at a time up to 6 weeks, yielded correlations of 0.92 at 2 weeks, 0.89 at 3 weeks, 0.85 at 4 weeks, 0.80 at 5 weeks, and 0.76 at 6 weeks.

## Discussion

The approach we have taken in the syndrome reporting system is to try to maximize the probability that any “signal” from the earliest stages of a bioterrorism or other public health event can be detected above the “noise” of normal clinical practice. The principal value of a syndromic surveillance system like the one described here is its ability to identify clusters of illness manifest by an unusual number of events, none of which individually differs appreciably from common respiratory, gastrointestinal, or other illnesses. Such nonspecific presentations might be the first sign of a widespread bioterrorism attack. They may also be the only routinely available clinical evidence of other important illness clusters, such as influenza or cryptosporidiosis, for which specific diagnostic tests are typically not performed. Even commonly available tests, such as x-rays, leukocyte counts, and sputum cultures are often not performed for lower respiratory illness with fever in an otherwise healthy patient, so syndromic surveillance can complement surveillance for individual cases of severe or unanticipated illness, which depend on detailed information about history, signs, symptoms, and diagnostic testing. Both syndromic and disease-specific surveillance systems are important components of any complete public health system.

To provide the best possible opportunities for effective intervention, an ideal surveillance system should gather timely, valid, and inexpensive data from a sufficiently large proportion of the population to detect events of interest in the region, and then process and present it to public health personnel in a form that enables efficient decision making. Important elements of such a syndromic surveillance system exist in the automated data generated by health plans and other parts of the health-care delivery system as part of routine operations. The system described here meets many of these criteria because it results from collaboration between academic investigators, health-care providers, and public health officials.

The automated medical records used here are well suited for surveillance of ambulatory-care encounters because the system is deeply integrated into the daily work of all clinicians and it is linked to both the provider payment and the membership systems. Although the data used in this system originate in a complete electronic medical record system, most of the syndromes are defined by diagnosis codes that are also available in other automated systems, including nurse hot lines and increasingly common same-day financial claims processing systems. Thus, several different kinds of data sources could contribute to an integrated surveillance network.

Time-series plots (Figure 4) provide some evidence that the data have validity as a measure of illness in the community, since the seasonal pattern is comparable with that in independently collected and validated hospital admission records for the same geographic region. The highest correlation, 0.92 at 2 weeks lag, implies that up to 85% of the variability of weekly hospital admission rates is predicted by variation in ambulatory-care admission levels 2 weeks earlier.

Although the principal focus of this system is identifying unusual patterns of apparently common conditions, it also ensures prompt reporting of any encounter with a diagnosis suggestive of a CDC category A bioterrorism agent. In practice, any clinician making one of these diagnoses would be likely to report such a case separately, but there is almost no marginal cost to implement or run this additional surveillance component.

The unadjusted counts and rates for each syndrome (Table 1) may be most useful in responding to a very large and widespread bioterrorism event or identifying expected events such as the advent of influenza in a community. In these cases, statistical refinement is unnecessary because those monitoring the system will see substantially elevated rates. We believe statistical inference will be most useful when the signal from an event is weak or restricted to a small geographic region. Many syndromes have large seasonal fluctuations, such as the well-known winter peak for lower respiratory disease. Individual census tracts also show substantial variability in daily syndrome episode rates, possibly associated with demographic and socioeconomic differences. The statistical model adjusts daily expectations to account for important sources of variation, so those parts of the report based on statistical models take large "expected" seasonal increases in illness into account (e.g., Figure 4). The sensitivity of the resulting system (Tables 1 and 2) in the face of expected variability appears to be much higher than more commonly advocated time-series based analytic approaches for public health surveillance (21).

Daily counts for each syndrome within single census tracts are usually zero, and as few as three to five health plan members affected would be unusual in a typical tract, depending on the month and day of week (Tables 1 and 2). To allow a rapid assessment of the distribution of illness in the region, we highlight the five extreme census tract counts for each syndrome in our daily reports, even though there is nothing unusual in any census tract on most days. An alerting system could easily be triggered when there is a sufficiently unusual cluster for any syndrome. The thresholds can be different for different syndromes, and they can be adjusted to accommodate any desired frequency of alerts. For example, in the absence of a period of heightened alert, public health authorities may wish to be notified when the daily count of syndrome episodes within any census tract attains a level that would only be expected to occur within the entire catchment area once every three or more months. Thus, users will be able to adjust the notification system to suit their needs in terms of the preferred balance of false-positive alerts against the risk of false negatives (no alert in the presence of an actual event of interest). This kind of information, which is being developed as part of this project, could be a useful supplementary source for other public health surveillance systems.

This reporting system includes strong protections of the privacy of individual patients’ health records, since routine reports contain only aggregated information. Existing clinical and administrative security protocols that control statutory or other authorized access to confidential patient data will apply when follow-up is requested by public health authorities.

One advantage of this system is that it takes advantage of the experience of ambulatory-care clinicians, who are likely to be among the first to encounter patients during the prodrome of any potential bioterrorism-related or other acute illness. In addition, the system imposes no additional reporting burden on clinicians, thus ensuring unbiased ascertainment of syndromes of interest that come to the attention of the practice. The data used here are already being gathered as part of the day-to-day practice of all participating clinicians. There is an initial cost for a system of this type because obtaining the data in a suitable form requires initial programming and testing, but subsequent processing requires relatively little additional expenditure and adds substantial value. All the technology used is widely available and inexpensive.

While any simplification inevitably hides some potentially important detail, we believe that in addition to making the reports more comprehensible, grouping the ICD-9 codes decreases the impact of variation in coding practices. This effect is particularly important since the earliest manifestations of an outbreak may be nonspecific. The fact that syndromic surveillance focuses on unusual counts of common events means that detection of a signal may not be greatly influenced by intensity of diagnostic testing performed, completeness of documentation in the medical record, or variation between physicians or health-care systems in the use of diagnostic terminology or assignment of ICD-9 codes. For example, we have shown that >90% of lower respiratory illness episodes are represented by only three of the 119 ICD-9 diagnosis codes included in the lower respiratory illness syndrome (8). As new ICD coding schemes are adopted, changes to the mapping used to translate code into syndrome will be required, but variation among tens of thousands of discrete individual codes is unlikely to have any major impact at the level of the broad syndromes used in our system.

This emphasis on broad groupings of diagnoses also supports the notion that different data sources, including automated medical records, nurse call centers, and transaction data, might be combined into an integrated surveillance system. Because the focus is on the acute illness that prompts a medical encounter, we expect that the performance characteristics will not be seriously affected by differences between automated data systems, for instance, in the number of diagnoses captured or in the method of assigning diagnosis codes. However, experience with additional systems will be required to elucidate these issues. To the extent that different systems yield similar discrimination of events of interest, it will be possible to integrate them at the regional level, to improve overall sensitivity, and at the national level, to allow coherent surveillance of the entire population.

While many types of data systems can contribute valuable surveillance information, appreciating the added value of more sophisticated data sources is also important. For instance, the availability of temperatures in the automated medical record system described here allows automated surveillance for influenza-like illness. The availability of automated laboratory test results and free text also provides opportunities to detect a wider array of conditions and to improve the specificity of detection of acute illness clusters. For example, anthrax surveillance might be limited to patients with fever and a lower respiratory illness syndrome.

An additional noteworthy feature of surveillance systems such as this one is the fact that they need not cover the entire population to identify at least some clusters of interest. The minimum proportion of the population that must be under surveillance to detect clusters of different sizes has not been determined, but our coverage of 5%-10% of the population of the region appears to provide useful information. Although a small fraction of ambulatory-care practices uses automated medical records, the effective population that would be covered by surveillance systems based on these automated records is substantial, including many of the major population centers in the country. Combining information from these sites with other information sources, such as those maintained by health plans or by hospitals, would rapidly provide at least some monitoring capability for a much larger overall population.

We can suggest additional methods for supplementing a surveillance system that counts syndromes encountered in ambulatory-care visits. Other sources of data, such as school and work absenteeism, over-the-counter medication sales, and even sales of products such as facial tissues and orange juice might contain potentially useful surveillance information. However, whether such data can be cheaply and efficiently gathered and processed and whether the data will yield valid and worthwhile signals remain to be demonstrated.

Many aspects of the current system will be improved with experience. The development of standardized grouping of ICD codes into syndromes is a priority to allow uniform reporting. A great deal of work remains in developing statistical methods capable of detecting different types of illness clusters, ranging from acute, localized increases (for instance, due to release of a toxic chemical agent) to more slowly emerging, widespread conditions, as might be expected from contamination of a water supply. The implementation described here demonstrates that existing electronic data developed in the course of routine medical care by a wide array of providers and health plans can yield substantial improvements in current public health capabilities for assessment of bioterrorism and other acute illness clusters.

## Appendix 1

We used this SAS code in fitting the generalized linear mixed model (GLMM) that generates the parameter estimates used in our reports. This SAS code relies on the GLIMMIX macro (17), which has been distributed by SAS (18) since version 6.12.%glimmix (data=test,procopt= noclprint covtest ,stmts=%str(class tract month dayofweek;model lri/pop=month dayofweek holiday day;random int/subject=tract solution type=un;),error=binomial);

The data set is structured to contain a row for each day in the historical period for each census tract. In the code, *lri* is the variable that contains the count for census tract *tract* on day *day*. *Pop* contains the number of subjects in the *tract* on that *day*. *Month* is the month of the *day*, *dayofweek* is the day of week of the *day*, and *holiday* indicates whether the *day* is a national holiday. Days are standardized to prevent numerical difficulties with computation.
